# Prevalence of hepatitis B virus surface antigen, associated risk factors, and liver enzyme abnormalities among individuals with diabetes in Aksum town public hospitals, Tigray, northern Ethiopia

**DOI:** 10.11604/pamj.2024.49.6.43263

**Published:** 2024-09-04

**Authors:** Mengstab Teklehaymanot Gebrelibanos, Krishna Chaithanya Karri, Tuem Gebre Abraha, Hailay Gebreyesus, Haftom Hadush Kidane, Mebrahtu Teweldemedhin Shfare

**Affiliations:** 1 Department of Chemistry, College of Natural and Computational Sciences, Aksum University, Aksum, Ethiopia,; 2 Department of Statistics, College of Natural and Computational Sciences, Adigrat University, Adigrat, Ethiopia,; 3 Department of Public Health, College of Health Sciences, Aksum University, Aksum, Ethiopia,; 4 Department of Medical Laboratory Sciences, College of Health Sciences, Aksum University, Aksum, Tigray, Ethiopia,; 5 Department of Medical Microbiology and Immunology, College of Health Sciences, Mekelle University, Mekelle, Tigray, Ethiopia

**Keywords:** Hepatitis B virus, diabetes mellitus, cross-sectional study, aspartate aminotransferase, alanine aminotransferase

## Abstract

**Introduction:**

hepatitis is an inflammatory disease of the liver; it is a major threat to public health and is more prevalent worldwide. Hepatitis B virus (HBV) is the leading cause of cirrhosis and hepatocellular carcinoma (HCC) with increasing mortality and burden of disease particularly in Asia and sub-Saharan Africa. Therefore, this study intended to assess the prevalence of HBsAg, associated risk factors, and liver enzyme abnormalities among individuals with diabetes mellitus (DM) in Aksum town public hospitals, Tigray, northern Ethiopia.

**Methods:**

a hospital-based cross-sectional study was conducted among 359 randomly selected individuals with diabetes mellitus in public hospitals of Aksum town from February 10 to May 10, 2021. A pre-tested structured questionnaire was used to collect the data. Data was entered into Epi-Data version 3.1 and analysis was made using the statistical software SPSS version 21 for Windows. Bivariate and multivariate Logistic regression model was applied to show association between the dependent and independent variables; P <0.05 and 95% confidence interval was considered for statistical significance.

**Results:**

in this study, 359 individuals with DM were included with a mean age (mean ± SD) of 46.44 ±16.58 years. The percentage of female participants was 44.3% (159/359). The prevalence of HBsAg among individuals with diabetes mellitus in Aksum town public hospitals was 12.8% (95% CI:8.9-17.0%). The associated risk factors were being employed [AOR:13.38, 95% CI 2.79-64.11; p<0.05], having history of multiple sexual partner [AOR:3.49, 95% CI 1.33-9.12; p<0.05], having history of body incision or piercing [AOR:3.80, 95% CI 1.12-12.90; p<0.05], liver enzyme abnormalities [AOR:6.90, 95% CI 2.17-21.94; p<0.005], and being single and widowed in marital status [AOR:4.42, 95% CI1.62-12.07; p<0.05].

**Conclusion:**

based on the HBsAg positivity, the prevalence of HBV among individuals with diabetes mellitus in this study area was high, as compared to the national findings. Therefore, integrated efforts should be made at the community and health facility level to raise awareness of the associated risk factors, and reduce the spread of HBV; targeted screening of HBV among people with diabetes is also important to minimize liver abnormalities.

## Introduction

Worldwide, about 400 million people are chronically infected while 1.5 million individuals die annually from HBV-related disease [1]. The virus can be transmitted sexually or by contact with contaminated blood or bodily fluids [2], and compared to Human immunodeficiency virus (HIV), It is 50-100 times more infectious [3]. Hepatitis B virus is recognized as an oncogenic virus that raises the possibility of hepatocellular cancer [4]. Infection with the virus can lead to acute hepatitis with discrete onset of symptoms or chronic with no symptoms [5]. The percentage of chronicity is approximately 5% in adulthood infections but it reaches 90% during childhood infections [5,6].

The highest prevalence of HBV is recorded in sub-Saharan Africa and East Asia, where between 5 and 10% of the adult population is chronically infected [7,8]. In most developing nations, including Ethiopia, HBV puts a significant burden on society due to frequent hospital stays, blood testing, and the high costs of treating chronic liver disease, liver failure, and loss of productivity [9].

Research has indicated that HBV, in addition to age, obesity, and lack of physical exercise, also plays a role in the development of diabetes mellitus (DM) by liver infection, inflammatory reaction, and by triggering pancreatic β-cell-specific autoimmunity, which in turn leads to DM [10,11]. Furthermore, people with diabetes are at high risk of HBV infection due to frequent hospitalization and repeated medical interventions [12,13].

The diagnosis of HBV is primarily by detecting hepatitis B surface antigen (HBsAg) [12]. However, there are few studies done in Ethiopia on the prevalence of HBV at a facility level; there is a scarcity of well-documented evidence regarding the prevalence of HBV among people with diabetes, particularly in the study area. Therefore, this study aimed to assess the prevalence of HBsAg, its associated risk factors, and liver enzyme abnormalities among individuals with DM in Aksum town public hospitals, Tigray, northern Ethiopia. The study was conducted taking into consideration the public health importance of HBV, and DM and the significance of the generated evidence towards the prevention of HBV, and improving diabetic care.

## Methods

**Study design and setting**: a hospital-based cross-sectional study was conducted from February 10 to May 10, 2021, in two public hospitals of Aksum town, which is located 1010 km North of Addis Ababa (the capital city of Ethiopia), and 250 km from Mekelle (the capital city of Tigray Regional State, northern Ethiopia). It has an elevation of 2131 m (6991 ft.) above sea level, annual average temperature of 18.3°C, annual rainfalls of 652 mm, and an area of 3247 km2. According to the town's administrative office (2018), it has about 70, 360 population, of which 53.54% were female and 46.45% were male. In the town, there are only two public Hospitals with diabetic care clinics, St. Marry General Hospital and Aksum University Comprehensive Specialized Hospitals.

**Study population**: the target population was all individuals with DM, visiting public hospitals of Aksum town Tigray, northern Ethiopia while the study population was selected individuals with DM. All confirmed individuals with DM aged 18 years and above attending the diabetes clinics of the two public hospitals were eligible for the study whereas those under antiviral therapy were not eligible for the study. The sample size was calculated based on the single-proportion formula [14]. The estimated prevalence of HBV among individuals with DM was not known in Tigray. Therefore, a p-value of 0.037 was taken from another study conducted in Woldya, Ethiopia [15]. Based on the assumption of the formula [14]:


$$n=\frac{Z_{\alpha /2}^{2}*P\left(1-p\right)}{d^{2}}$$

**Figure 1:**
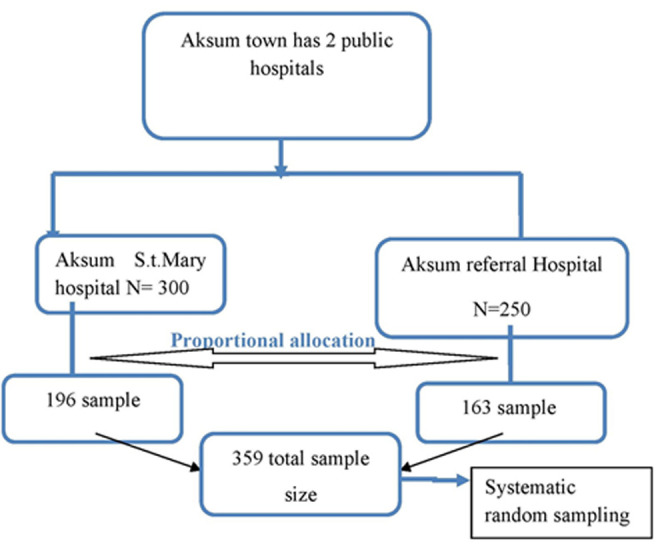
schematic presentation of the sampling procedure

Where: n is the sample size, considering the 95% confidence level, α is 0.05 (the level of significance) and the value of Z at α/2 is 1.96; d is the margin of error (we considered 2%). Accordingly, n =342; with 5% contingency the final sample size was 359. During the sampling procedure, considering the patients flow at each health facility, the total sample size was distributed using the technique of population proportion to size allocation (PPS). Systematic random sampling technique was employed to select each study subject where every second patient was selected and included in the study (Figure 1).

**Data collection:** a pre-tested structured questionnaire, translated into Tigrigna (local language), was used to collect the demographic and risk factors related data [16]. Recruited clinical nurses filled out the questionnaire through direct interview of the study participants. After completion of the questionnaire, trained laboratory technologists collected adequate specimens (paired 5-10ml of blood of venous blood) from the forearm of each stud subject following aseptic techniques. The whole blood was centrifuged at 3000rpm for 5 minutes to get a serum sample in the hospital; the serum was tested for hepatitis B surface antigen (HBsAg) using rapid screening test kits (CIKBiotech). The test kits had a sensitivity and specificity of 99.8% and 99.5% respectively.

A liver enzyme test was done using an automated clinical chemistry analyser to measure the extent of liver enzyme elevation/abnormality i.e aspartate transaminase and alanine transaminase (ALT and AST) and the corresponding reference values were determined based on the manufacturer directions in the reagent leaflets (Mindray BS-200E Afro Germany, human Reagent). Additionally, blood glucose level was measured using a glucometer (i-Qare DS-W blood glucose meter, 139 Taiwan). To ensure the quality of the data collection, five percent of the questionnaire was pre-tested among DM patients in Adwa town and the questionnaire was revised accordingly. Two days of training were given to the data collectors (nurses and laboratory technologists). Supervisors immediately for legibility or other errors confirmed the filled questionnaires. Test kits and reagents were pre-checked for expiry date, damage, and storage problems. Quality controls were done simultaneously to monitor the quality of laboratory results Standard operating procedures were followed during specimen collection, handling, transportation, and laboratory investigation [16].

### Definitions

**Dependent variable:** HBsAg serostatus: determined based on the detection of HBsAg in serum.

Independent variables: the independent variables considered in this study include socio-demographic variables (age, gender, residence, marital status, educational level, and occupation). Harmful traditional practices (body piercing (at multiple sites body incision, ear piercing, tattooing), uvula cutting, and circumcision). A multiple-site body incision is a traditional practice that parents do on their children in some parts of the region with the intention/belief to remove blood in edema or any site body swelling. Clinical factors (history of blood transfusion, history of needle stick injury, history of hospitalization, history of teeth extraction, having multiple sexual partners, history of chronic liver disease, liver enzyme abnormality, and blood glucose level). Here, liver enzyme abnormality was defined as an elevation of serum Aspartate aminotransferase and Alanine aminotransferase levels from the normal ranges.

**Statistical analysis:** data was entered into Epi-Data version 3.1 and analysis was made using the statistical software SPSS version 21 for Windows. In the SPSS, descriptive summaries were used to crosscheck any missing values. Quantitative variables (age, enzyme levels and glucose level) were grouped to categorical variables. Bivariate and multivariate logistic regression model was applied to show the association between the dependent and independent variables. Firstly, each categorical independent variable was tested for association with the dichotomous dependent variable (HBsAg serostatus). All variables that showed an association at P<0.05 in the bivariate analysis were reanalysed simultaneously in a multivariate analysis to adjust confounders. Finally, the statistical association was declared considering P<0.05 and 95% confidence interval. Findings were presented using statistical tables and were discussed in detail with related studies.

**Ethical consideration:** ethical approval was obtained from Aksum University Comprehensive Specialized Hospital´s (AKU, CHS and CSH) institutional review board (reference number: IRB 233/2020). Official permission was obtained from each hospital. Written informed consent translated into Tigrinya (local language), was explained to each study participant before data collection; consented candidates were not forced to give consent. No personal identifiers were mentioned during data collection, identification was based on a unique identification number given for the particular questionnaire and corresponding blood sample.

## Results

**Socio-demographic characteristics of the study participants:** in this study, 359 clients were participated or interviewed with a response rate of 100%. The mean age (mean ± SD) of the participants was 46.44 ±16.58 years, range (19-76 years). The number of male participants was 200 (55.7%); regarding marital status about 267 (74.4%) of the respondents were married. About 175 (48.7%) of the respondents were unable to read and write. Furthermore, about 188 (52.4) of the respondents were urban residents (Table 1).

**Table 1:** socio-demographic characteristics of individuals with DM attending the diabetic clinic in Aksum town public hospitals, northern Ethiopia, 2021

Variables		Frequency [n= 359]	Percentage [%]
**Age**	19-34	40	11.1
	35-49	80	22.3
	50-64	112	31.2
	≥65	127	35.4
**Sex**	Male	200	55.7
	Female	159	44.3
**Marital status**	Single	62	17.3
	Married	267	74.4
	Divorced	15	4.2
	Widowed	15	4.2
**Educational status**	Unable to read and write	175	48.7
	Primary school	85	23.7
	Secondary school	53	14.8
	Diploma and above	46	12.8
**Occupation status**	Farmer/housewife	147	40.9
	Merchant	91	25.3
	Employed	48	13.4
	Unemployed	73	20.3
**Residence**	Rural	171	47.6
	Urban	188	52.4

**Figure 2:**
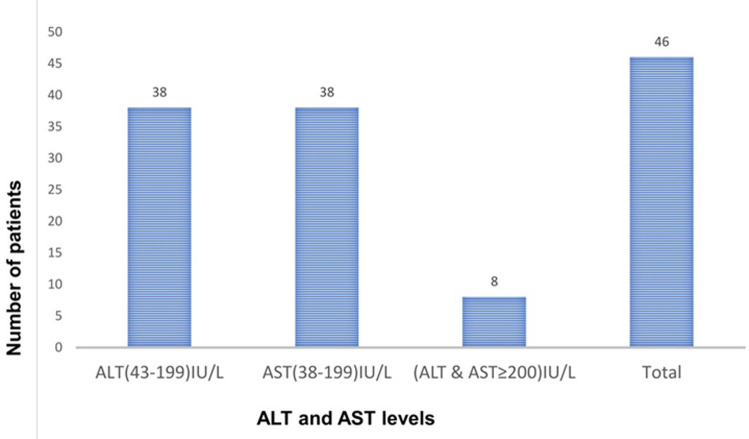
aspartate transaminase and alanine transaminase levels of HBsAg positive individuals with diabetes mellitus attending the diabetic clinic in Aksum town public hospitals, northern Ethiopia, 2021

**Harmful traditional practices, clinical characteristics, and HBsAg positivity among individuals with diabetes mellitus:** based on the serostatus of HBsAg, the prevalence of HBV in this study was 12.8% (95% CI 8.9-17.0%). The prevalence of uvula cutting practice, circumcision, multiple sexual partners, and traditional body skin incisions in this study were 245 (68.2%), 210 (58.5%), 54 (15%), and 157 (43.7%), respectively (Table 2). Concerning the clinical characteristics, the most prevalent observations were a history of hospitalization/invasive procedures (12.3%), need stick injury (13.9%), a history of blood transfusion (20.9%), and liver enzyme abnormalities (22.3%) (Table 3). According to the liver function test, all of the HBV-positive participants had abnormal ALT and AST levels among which 8/46 (17.4%) were acute infections (AST and ALT: ≥ 200 IU/L) (Figure 2).

**Table 2:** harmful traditional practices of individuals with diabetes mellitus attending the diabetic clinic in Aksum town public hospitals, northern Ethiopia, 2021

Variables		Frequency [n=359]	Percentage [%]
History of uvula cutting	No	114	31.8
	Yes	245	68.2
History of circumcision	No	149	41.5
	Yes	210	58.5
History of body incision or piercing	No	202	56.3
	Yes	157	43.7
History of multiple sexual partners	No	305	85.0
	Yes	54	15.0

**Table 3:** clinical factors of individuals with diabetes mellitus attending the diabetic clinic in Aksum town public hospitals, northern Ethiopia, 2021

Variables		Frequency [n=359]	Percentage [%]
Test for HBsAg	No	313	87.2
	Yes	46	12.8
History of teeth extraction	No	316	88.0
	Yes	43	12.0
History of blood transfusion	No	284	79.1
	Yes	75	20.9
History surgery, hospitalization or invasive procedures	No	315	87.7
	Yes	44	12.3
History of needle stick injury	No	309	86.1
	Yes	50	13.9
History of chronic liver disease	No	342	95.3
	Yes	17	4.7
Liver enzyme abnormalities (AST or ALT)	Normal	279	77.7
	Abnormal	80	22.3
Glucose level	80-100	32	8.9
	101-125	37	10.3
	>126	290	80.8

**Bivariate and multivariate analysis of factors associated with positive HBsAg:** in the univariate analysis, age, marital status, occupational status, history of multiple sexual partners, history of body incision or piercing, history of teeth extraction, history of blood transfusion, history of needle stick injury, and liver enzyme abnormality were found to be significantly associated with positive HBsAg at p<0.05. After the multivariate analysis, the factors associated with positive HBsAg were single in marital status [AOR:4.42, 95% CI1.62-12.07; p<0.05], occupation [AOR:13.38, 95% CI 2.79-64.11; p<0.05], history of multiple sexual partners [AOR:3.49, 95% CI 1.33-9.12; p<0.05, history of body incision or piercing [AOR:3.80, 95% CI 1.12-12.90; p<0.05, and liver enzyme abnormality [AOR: 6.90, 95% CI 2.17-21.94; p<0.05) (Table 4).

**Table 4:** factors associated with positive HBsAg among individuals with diabetes mellitus attending public hospitals of Aksum town Tigray, northern Ethiopia, 2021

Variables		Test results for HBsAg		Unadjusted OR (95% CI)	Adjusted OR (95% CI)
		Negative (%)	Positive (%)		
**Age in years**	19-34	34(85)	6(15.0)	2.18(0.65-7.24)	3.40(0.66-17.51)
	35-49	74(92.5)	6(7.5)	1	1
	50-64	92(82.1)	20(17.9)	2.68(1.02-7.02)*	2.64(0.70-10.01)
	≥65	113(89.0)	14(11.0)	1.53(0.56-4.16)	2.26(0.55-9.30)
**Marital status**	Single	43(69.4)	19(30.6)	4.92(2.46-9.85)*	4.42(1.62-12.07)**
	Married	245(91.8)	22(8.2)	1	1
	Divorced	12(80.0)	3(20.0)	2.78(0.73-10.61)	2.04(0.28-14.88)
	Widowed	13(86.7)	2(13.3)	1.71(0.36-8.08)	2.16(0.28-16.66)
**Occupation status**	Farmer/housewife	140(95.2)	7(4.8)	1	1
	Merchant	76(83.5)	15(16.5)	3.95(1.54-10.10)*	4.08(1.07-15.64)**
	Employed	39(81.3)	9(18.8)	4.62(1.62-13.18)*	13.38(2.79-64.11)**
	Unemployed	58(79.5)	15(20.5)	5.17(2.01-13.35)*	2.50(0.63-9.99)
**History of multiple partners**	No	284(93.1)	21(6.9)	1	1
	Yes	29(53.7)	25(46.3)	11.66(5.82-23.35)*	3.49(1.33-9.12)**
**History of body incision or piercing**	No	196(97.0)	6(3.0)	1	1
	Yes	117(74.5)	40(25.5)	11.17(4.60-27.14)*	3.80(1.12-12.90)**
**History of teeth extraction**	No	283(89.6)	33(10.4)	1	1
	Yes	30(69.8)	13(30.2)	3.72(1.77-7.82)*	1.74(0.50-6.02)
**History of blood transfusion**	No	253(89.1)	31(10.9)	1	
	Yes	60(80.0)	15(20.0)	2.04(1.04-4.02)*	1.92(0.63-5.91)
**History of needle stick injury**	No	282(91.3)	27(8.7)	1	
	Yes	31(62.0)	19(38.0)	6.40(3.20-12.82)*	2.23(0.85-5.82)
**Liver enzyme Elevation (AST, ALT)**	Normal	264(94.6)	15(5.4)	1	1
	Abnormal	49(61.3)	31(38.8)	11.14(5.60-22.15)*	6.90(2.17-21.94)**

Note: 1: referent group; HBsAg: hepatitis B surface antigen. Where: OR (odds ratio); CI (confidence interval); values written in bold and with star (*) and (**) show statistically significant association (p value<0.05) respectively; ALT (Alanine aminotransferase) =0-42IU/L: normal; ALT≥43: abnormal; AST (Aspartate aminotransferase)=0-37IU/l: normal; AST≥38IU/L: abnormal

## Discussion

This facility-based cross-sectional study tried to assess the prevalence of HBsAg and its risk-associated factors among individuals with DM in Aksum town public hospitals in central Tigray, northern Ethiopia. In this study, the prevalence of Hepatitis B virus based on HBsAg seropositivity was 12.8%, [95% CI 8.9-17.0%]. Occupational status, history of multiple sexual partners, history of body incision, liver enzyme abnormalities and history of marital status were significantly associated factors with positive HBsAg. In the Ethiopian context, there is only one study to compare with; our finding is higher compared with the only one previous cross-sectional study conducted among individuals with DM in Woldya General Hospital, Ethiopia 3.7% [15]. The high prevalence of HBsAg observed in the current study might be due to occupational status, history of multiple sexual partners, history of body incision, and history of marital status revealed as significant risk factors. Similarly, the prevalence of HBV in the current study is also higher than the findings from studies conducted among individuals with DM in different parts of the world; Ghana 5.5% [17], Congo 3.4% [18], Turkey 5.1% [19] and that of Iraqi 2.13% [20]. However, a similar finding has been reported in a cross-sectional study conducted in China 13.5% [21].

As we highlighted earlier, because there are limited studies on the same group in the local and national context, we tried to compare the prevalence of HBV with other study groups. Hence, the prevalence of HBV in this study was found to be in line with the prevalence of HBV among patients scheduled for surgery in Hawassa, south Ethiopia 9% [22]. However, the current finding is higher than the prevalence of HBV in several studies conducted among other study groups in Ethiopia; among healthcare workers (5%), among HIV positive individuals (5%), blood donors (4%) [23], and pregnant women (6.6%) [24]. Additionally, a higher prevalence (22.3%) was reported among individuals with chronic hepatitis in southeast Ethiopia [25].

In this finding occupational status was significantly associated with positive HBsAg. The possible reason for occupational status may be due to having multiple sexual partners, high occupational exposures to blood or body fluids from the source of patients, materials, and any infected body [26]. In this finding, study subjects who had a history of multiple sexual partners were significantly associated with positive HBsAg as compared to those who did not have a history of multiple sexual partners. The possible reason may be due to blood, semen, or vaginal fluid contact during sexual intercourse with infected partners; a study indicated that the prevalence of HBV infection was 6% for those who have fewer than five sexual partners versus 21% for those who have five or more sexual partners [27]. History of multiple sexual partners was also indicated as a significant predictor of seropositivity for HBV in other studies conducted among individuals scheduled for surgery at Hawassa University [22], delivering women in Addis Ababa [9], and pregnant women attending routine antenatal care in Arba Minch [28]. Similarly, the odds of seropositivity of HBV were 17.38 times higher among pregnant mothers with a history of multiple sexual partners according to a facility-based cross-sectional study conducted in Gambella [29]. Hence, the higher odds of positive HBsAg among those single in their marital status in the current study might also be attributed to active sexual practices and multiple sexual partnership behaviors.

In the current study history of body incision has been implicated to have a significant association with the prevalence of HBV among Individuals with DM. The odds of seropositivity for HBsAg were higher among patients who had a history of body incision. The possible reason for this finding may be due to the usage of unsterilized material during pricing and unsafe socio-cultural tattoos or traditional healing mechanisms done in the study area. Likewise, body incision/piercing was a significant predictor in other studies conducted among pregnant women in Addis Ababa [30], Bahir Dar [24], Deder Hospital [31], and among health professionals in Gondar Hospital, northwest Ethiopia [32].

The other finding concerning the risk factors is liver enzyme abnormality (elevated AST or ALT results). The HBsAg positivity was higher among patients who had liver enzyme abnormalities (AST or ALT) as compared to their counterparts. These results are in line with the previous studies conducted among individuals with DM in Taiwan [33] and Turkey [19]. The ALT and AST values imply that most of the HBsAg-positive individuals with DM had chronic HBV infection whereas 17.4% (ALT and AST values ≥200IU/L) of them were having either an acute infection or a replicative chronic infection. During acute HBV infection or the replicative phase of chronic HBV infection, a massive increase in enzyme activity is expected due to the high degree of viral infectivity, cell-mediated immune response, and liver injury [34]. During reactivation of HBV, the marked increase in viral replication is accompanied by the reappearance of inflammatory necrosis in the liver, and a 10-fold elevation in ALT level is indicative of acute exacerbation of flare of HBV [35].

The findings of the current study imply that individuals with DM were majorly affected by chronic HBV infection with multiple risk factors. Considering the infectiousness, capability of horizontal and vertical transmission and potential carcinogenesis, the current findings urge for massive and targeted interventions. The current study has strengths in that it addressed a population and a virus of great local and global attention, and explored the harmful factors including the multiple body site incisions grounded in the study area. This generated evidence can fill the information gap, which can be employed for public interventions including awareness creation, targeted vaccination, and screening. As a limitation, this prevalence study did not assess other possible reasons for the elevation of liver enzymes including drug interactions, and herbal medications in society though the liver enzyme abnormality has shown a statistically significant association (adjusted odds ratio: 6.90) with the outcome variable.

## Conclusion

The prevalence of HBV, based on the positivity for HBsAg, among individuals with DM in this study area is high as compared to the national findings. Occupational status, history of multiple sexual partners, history of body incision, liver enzyme abnormalities, and marital status were found to be significantly associated with the prevalence of HBV in this study. The findings on ALT and AST values implicate that most individuals with DM were chronically infected. Therefore, integrated efforts should be made at community and health facility levels to raise awareness of the associated risk factors, and reduce the spread of HBV; targeted screening of HBV among people with diabetes is also important to minimize liver abnormalities.

### What is known about this topic

- *Hepatitis B virus (HBV) is recognized as one of the oncogenic viruses;*
- *It is the leading cause of cirrhosis and hepatocellular carcinoma (HCC);*
- *HBV-associated burden of disease is high in Asia and sub-Saharan Africa*.

### What this study adds

- *The study addressed a new study area with regard to the prevalence of HBV among individuals with DM, and associated factors;*
- *High HBsAg positivity rates were found in the current study among individuals with DM compared to the national findings in Ethiopia;*
- *The study revealed additional risk factors including multiple body incisions that are traditional healing practices rooted in the study area*.
